# The presence of bacterial microcolonies on the maxillary sinus ciliary epithelium in healthy young individuals

**DOI:** 10.1371/journal.pone.0176776

**Published:** 2017-05-02

**Authors:** Monika Morawska-Kochman, Krzysztof Marycz, Katarzyna Jermakow, Kamil Nelke, Wojciech Pawlak, Marek Bochnia

**Affiliations:** 1 Department of Otolaryngology, Head and Neck Surgery, Medical University, Wroclaw, Poland; 2 Department of Hygiene and Ichthyology, Electron Microscope Laboratory, Environmental and Life Science University, Wroclaw, Poland; 3 Department of Microbiology, Medical University, Wroclaw, Poland; 4 Department of Maxillofacial Surgery, Medical University, Wroclaw, Poland; Beijing Tongren Hospital, CHINA

## Abstract

**Objective:**

The aim of this cross-sectional in vitro study was to evaluate the mucosal surfaces of healthy maxillary sinuses, explore different forms of bacterial microorganism colonies present on the mucous membrane, and determine a mucosal surface area they occupy.

**Methods:**

Samples of the maxillary sinus mucosa were collected from 30 healthy patients (M = 11; F = 19). The material was obtained during the Le Fort I osteotomy performed during corrective jaw surgery. The morphological and morphometric analysis of sinus mucosa and bacterial film that was grown on it was performed using scanning electron microscopy (SEM) as well as imaging software.

**Results:**

Scanning electron microscopy analysis showed the presence of different bacterium and bacteria-like structures in all the analyzed samples. In most cases, the bacterial film was mostly composed of diplococci-like and streptococci-like structures on the mucosa of the paranasal sinus. In any case, the mucous layer did not cover the whole lining of the evaluated sample. Each colony consists of more than 20 single bacterial cells, which has grown in aggregates.

**Conclusions:**

Under the conditions of normal homeostasis of the body, the maxillary sinuses present diverse bacterial colonization. The bacteria are dispersed or concentrated in single microcolonies of the biofilm on the border of the mucous covering the ciliary epithelium. There is no uniform layer of the biofilm covering the mucosa of the maxillary sinuses. Because the biofilm is detected on healthy individuals sinus mucosa, the clinical question if it may become pathogenic is unclear and require an explanation.

## Introduction

The bacterial biofilm is typically defined as the concentration of microorganisms superficially associated and surrounded by extracellular polymeric substances (EPS). Its creation, as the microbial survival strategy, is characteristic for many pathogenic and non-pathogenic bacterial species [1]. It is currently believed that the biofilm is responsible for some chronic inflammations [2–7, 8].

Clinical biofilm-associated infections (BAI) are recognized also as a greater therapeutic problem than infections caused by the planktonic form of bacteria. *In vitro* studies on the biofilm are then typically focused on the research of pathogenic strains. Microbiological identification of the biofilm responsible for BAI is especially difficult due to the need for differentiation from non-pathogenic colonizing flora [3]. It should also be noted that pathogenic organisms, such as *Pseudomonas aeruginosa*, *Haemophilus influenzae*, *Streptococcus pneumoniae*, or *Staphylococcus aureus* can be found in patients without active symptoms of the disease [9–11]. Usually, colonization is defined as the presence of bacteria on the mucous membrane, and the lack of the inflammatory response distinguishes it from an infection [12].

The Hall-Stoodley and Stoodley studies indicate that the establishment of the biofilm may be a form of a bacterial carrier state and its presence is in contrast to the infectious form of the bacteria [1].

This problem is particularly difficult because of the small number of clinical trials on the biofilm in healthy subjects. Therefore, identifying the consequences of the presence of opportunistic bacteria on the mucosa in the carriers in relation to the reaction to pathogenic bacteria requires further studies.

However, the bacteria film in contrast to typical biofilm might be defined by the presence of bacteria, that growth in colonies without inducing the inflammatory response. Thus, the aim of the study was to evaluate the mucosal surfaces of the healthy maxillary sinuses (without any history of recent acute sinus inflammations or chronic inflammation in the past), to identify different forms of bacterial microorganisms which could, under certain conditions, become opportunistic or pathogenic and determine a mucosal surface of the area they occupy.

## Materials and methods

### Ethical considerations

The protocol for the study was granted approval by the Bioethics Committee at the local Medical University (KB-545/2015). Each patient signed an individual consent form and decided freely to participate in this study.

### Patients

Patient recruitment was performed at the Department of Maxillofacial Surgery of the Medical University Hospital.

The group of subjects included generally healthy young people (n = 30), 19 women (63.34%) and 11 men (36.66%) (the mean age ratio was 21 years; SD = 5,69; MIN-18 y. MAX- 40 y.). The patients were scheduled for planned orthognathic surgery. Qualification for the surgery included computed tomography (CT), facial and jaw radiographs followed by laboratory tests performed immediately before the surgery which included, among others, basic inflammatory markers (blood cell count, CRP- C-reactive protein, procalcitonin and fibrinogen levels). Additional endoscopic evaluation of the nasopharynx and the oral part of the pharynx were performed.

The age range of the patients included in the study was between 18 to 40 years old, due to the nature of the orthognathic surgery. The surgical correction with a Le Fort osteotomy included patients who were at least 15 years old. There is not an upper age limit, but adults over the age of 40 rarely decide to have such procedures done. The inclusion criteria for the study group covered those with no medical history of paranasal sinus conditions or other diseases.

Two basic diagnostic procedures, performed only in the month preceding the surgery, consisted of endoscopic and CBCT (cone-beam CT) evaluation of the nasal cavities. Patients without any symptoms or clinical manifestations of sinus diseases took part in the study.

A nasal swab from each participant was taken 14 days before the planned surgery. Microorganisms, such as *Staphylococcus epidermidis*, or *Staphylococcus aureus* were considered here as relatively non-pathogenic flora in accordance with the available literature [9–11].

The criteria for exclusion from the study group included both rhinitis and sinusitis (inflammation or allergy), and antibiotic therapy or steroids (topical or general) taken within four weeks prior to the surgery. Each time lesions within the sinuses were excluded. Pathological flora in swabs from the nasal mucosa or pathological lesions of the paranasal sinus mucosa were the main exclusion criteria from the study. Also, any history of surgical treatment or fractures in the nose and (or) paranasal sinuses were taken into consideration. Patients lacking adequate clinical examinations consisting of endoscopic and radiological imaging were excluded from the study group.

### Surgery

Samples of the maxillary sinus mucosa were collected from 30 young healthy patients (the mean age ratio was 21 years; SD = 5,69) who were operated on because of various forms of dentofacial deformations during orthognathic surgery procedures. The procedures included the maxillary bone corrective osteotomy technique described as the Le Fort I osteotomy. With access for the typical Le Fort I osteotomy, a surgeon took samples from the maxillary sinus mucosa surplus (mean size 3x5 mm). The material was collected immediately after cutting the bony area of the anterior and lateral maxillary walls and the surgical breakage of the pterygoid process. This osteotomy manoeuvre enabled uncovering the mucosa of the upper posterior part of the sinus. The tissue specimens were prepared for microscopy evaluations directly after their removal. In addition, swabs were taken from both the maxillary sinus and nasal mucosa intraoperatively and were immediately sent to a microbiology lab for further analysis. We suspected anaerobic organisms so all patients’ specimens were protected from the toxic effect of oxygen and were transported to the laboratory with minimal delay. Samples were inoculated onto Becton Dickinson’s prepared media: Columbia Agar with 5% Sheep Blood, MacConkey II Agar, Schaedler Agar with Vitamin K1 and 5% Sheep Blood, Chocolate II Agar, Sabouraud Dextrose Agar with Chloramphenicol and Tryptic Soy Broth. Cultures were incubated at 35–37°C for 24–72 h on solid agar media, and for 21 days in liquid media.

The organism’s microscopic morphology and Gram staining characteristics were the first screening step of identification. Bacteria were identified using culturing and measuring growths of individual microorganisms. Each cultured bacterium was identified by biochemical test in the automatic system BBL Crystal (Becton Dickinson^®^). Depending on the bacteria species, the tests used were as follows:

- BBL Crystal GP—for the identification of Gram-positive *Cocci* and *Rods*,
- BBL Crystal N / H—to the identification of strains of *Neisseria*, *Haemophilus*
- BBL Crystal ANR—for the identification of anaerobic bacteria,
- BBL Crystal E / NF for the identification of Gram-negative *Enterobacteria* and non-fermenting and also one-off tests such as the production of indole and oxidase as well as the presence of catalase.

The cultures and all the diagnostic tests were performed according to the principles of microbiological diagnostics.

### Sample preparation for microscopy

Directly after swabs were taken, the samples of sinus mucosa were fixed in 2.5% glutaraldehyde in phosphate buffer (137 mM NaCl, 2.7 mM KCl, 10 mM Na_2_HPO_4_, 2 mM KH_2_PO_4_, pH 7.2–7.4), transferred within 24 hours, and stored for scanning electron microscopy (SEM) analysis at 4°C overnight. After fixation, the samples were washed three times for 10 minutes in distilled water. For dehydration, 50% ethanol was added to the samples and incubated at room temperature for 10 minutes. The process was repeated with 60%, 70%, 80%, 90%, and 98% ethanol, and were afterward suspended in 100% ethanol and left to air-dry overnight. Then, the samples were placed on a SEM stub, coated with gold using ScanCoat6 (Edwards), and observed by means of an SE1 detector, at 10 kV filament tension (SEM, Zeiss Evo LS 15).

### Identification of the presence of the biofilm

Evaluation of each specimen under a scanning microscope (SEM) Zeiss Evo LS 15 (Carl Zeiss NTS GmbH- Germany) was carried out twice, by experienced investigators, who were blinded to the samples.

The process of identifying bacterial films was partially performed by means of specific biofilm diagnosing criteria presented by Parsek and Singh, different from clinical ones as those are related to biofilm infections [13]. According to the authors, pathogenic bacteria were associated with a surface. The direct examination of tissue demonstrates aggregated cells in cell clusters encased in a matrix.

## Results

Scanning electron microscope investigations revealed the presence of bacterial film on the surface of maxillary sinus mucosa in 30 patients. Moreover, microbiological examinations of specimens taken from study participants revealed the presence of various types of aerobic and anaerobic bacteria in 28 cases (93.34%) out of 30 studied samples.

All samples had mixed flora. In total, 41 different microorganisms were isolated. The most frequently found microorganism was *Streptococcus spp*. in over 90% of all samples, while *Propionibacterium acnes* were present in 29,2% of samples, and *Staphylococcus spp*. was present in 17% of the samples.

**Fig 1:**
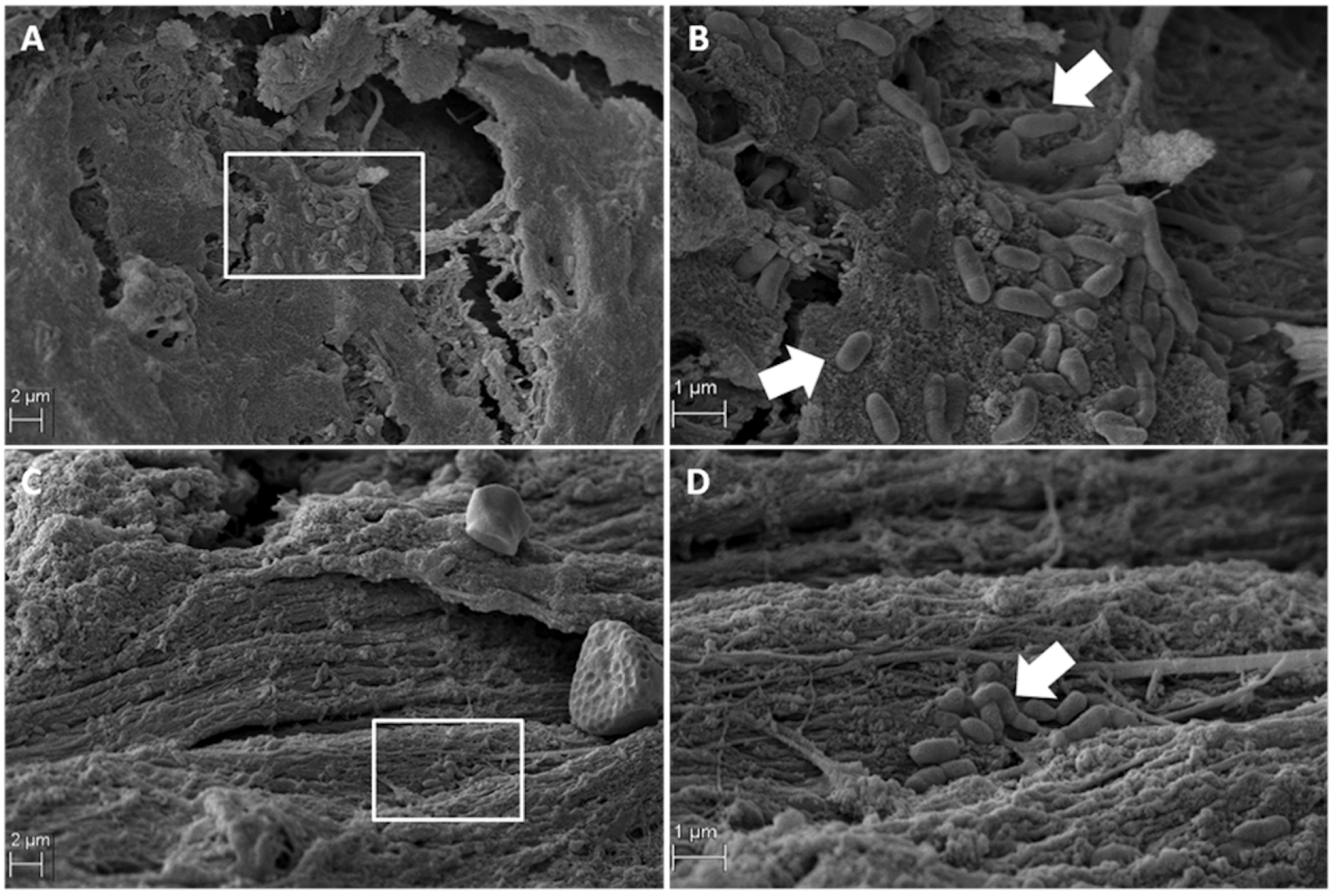
Scanning electron microscopy (SEM) images of biofilm architecture seen on the mucosal surface of the healthy paranasal sinus mucosa. The rod-shaped Staphylococcus epidermidis-like colonies (Fig 1B and D, white arrows) at the base of the cilia, usually located in the peripheral region of the mucosa (Fig 1A and C), (A, C-original magnification ×500. B, D- original magnification ×2000).

**Fig 2:**
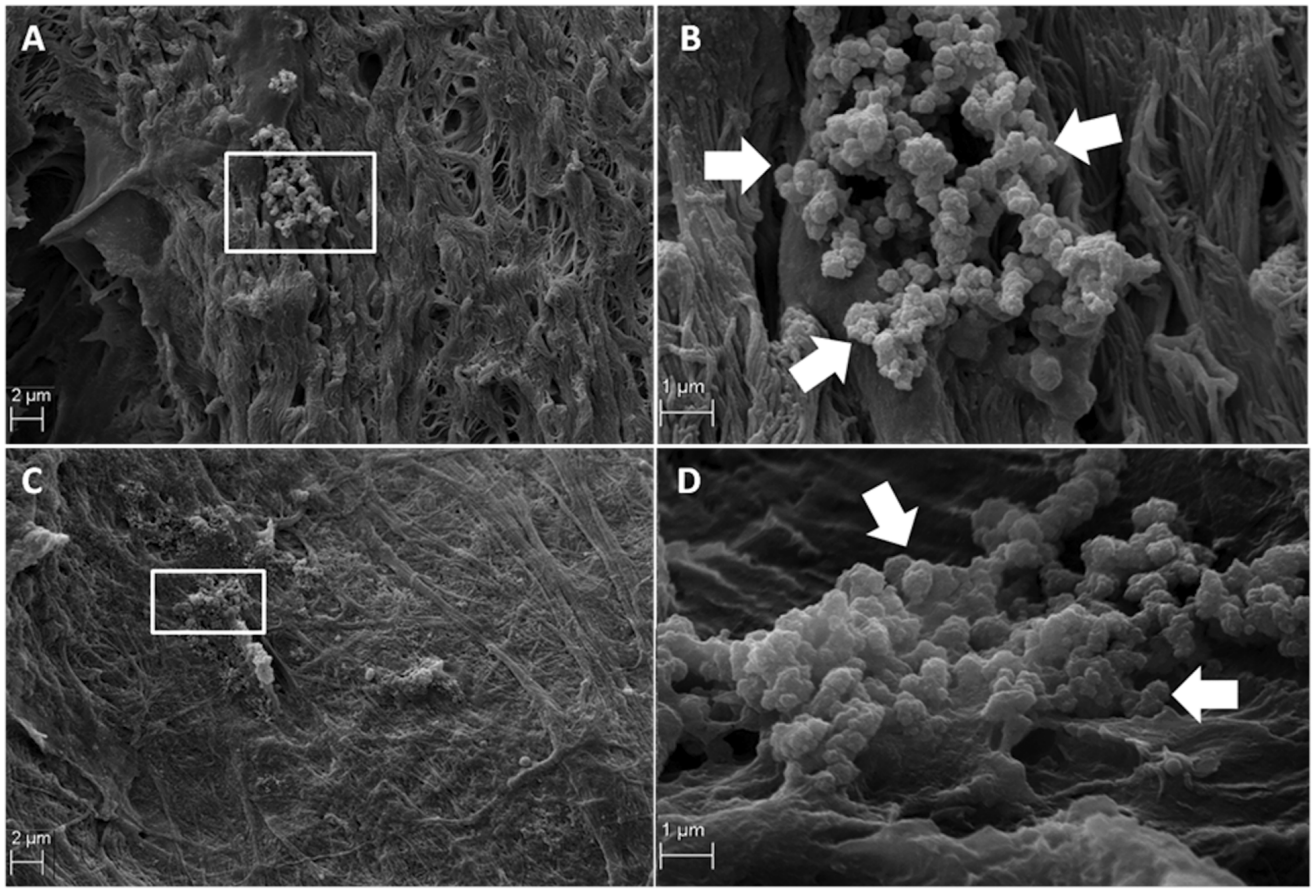
Scanning electron microscopy images of biofilm architecture seen on the mucosal surface of the healthy paranasal sinus mucosa. Spherical structures related to Haemophilus influenzae (Fig 2 B and D, white arrows). A single bacterial microcolony (Fig 2 A and C)—(A, C-original magnification ×500. B, D- original magnification ×2000).

Scanning electron microscopy analysis showed that the mucous layer has a thickness of 200 nm (± 40), which is covered up to 5% of the surface of each sample. The analysis showed the presence of bacteria-like microcolony structures in all analyzed samples. Scanning electron microscopy revealed the presence of the biofilm mostly composed of diplococci-like and streptococci-like structures in the paranasal sinus mucosa obtained from different patients. A lot of rod-shaped *Staphylococcus epidermidis*-like colonies (Fig 1B and 1D, white arrows) were clearly seen at the base of the cilia, usually located in the peripheral region of the mucosa (Fig 1A and 1C). Moreover, the authors observed spherical structures similar to *Haemophilus influenzae* in shape and size (Fig 2B and 2D, white arrows). These bacteria were characterized by an elongated shape and defined as apical regions. This kind of microcolony was usually present on the surface of the mucosa but was also identified on the regular cilia. A single bacterial microcolony typically occupied up to 1% of the test sample surface (Fig 2A and 2C). Smears from the sinus of the patient, presented in photographs 1 and 2, revealed *Staphylococcus epidermidis* and *Streptococcus sobrinus*. The second presented patient, apart from streptococci and staphylococci, also had anaerobic bacteria *Propionibacterium acnes*, *Veillonella* sp., and *Peptostreptococcus* sp. The morphometric analysis revealed the presence of 22 (±2) and 43 (±8) *Streptococci* and *Cocobacilar* like microcolony, respectively. The method of morphometric analysis was previously described by Marycz, et al. [14]. Each colony consisted of more than 20 single bacterial cells, that had grown in aggregates. These clearly indicate the existence of a bacterial-like microcolony on maxillary sinus mucosa.

## Discussion

The human microbiome is the total number and diversity of microbes found in and on the human body. The ability to cultivate organisms from tissues and clinical samples like swabs were the gold standard for identification of normal flora and pathogens. However, molecular detection methods, based on DNA sequencing, indicates that the human body contains a far greater bacterial diversity than previously recognized [3].

Modern research, using new specific genetic detection techniques, focuses on interactions between a human and microorganisms residing within and on the surface of our bodies [15]. These findings reveal that the presence of microbiomes could contribute to the pathogenesis of many illnesses (e.g., asthma, allergy, obesity, diabetes) [15–16]. The Human Microbiome Project was created in order to study the microbiome in human health [17]. In the microbiome field, many variables of interest, such as diet or lifestyle influence, are not yet fully understood. However, modern research on microbiota provides a lot of information about the organisms colonizing sinus mucosa in healthy individuals, even though it does not allow to determine the size and location of biofilm structures. Corsterton found that bacteria in a humid environment were organized communities of aggregated cells [18]. This ability to adapt allows bacteria to survive in a variety of conditions, facilitating survival at a given population level [1]. The process of the biofilm formation proceeds from planktonic bacteria, through formation of microcolonies, to a mature structure creating a matrix [8]. Because the form of bacterial aggregation and biofilm formation structures might be correlated to host-pathogen interactions [1], it appears that the definition of its structure will distinguish significant pathogenic biofilms from nonpathogenic microbial colonization.

Host-microbial balance might play important role in an initiation of inflammation of sinus mucosa. Most studies focused on patients suffering of chronic sinusitis with biofilm layer. It was shown that active disease could be associated with decreased levels of the antimicrobial proteins (i.e. laktoferrin) or failure pattern recognition of receptor pathways (i.e. bitter taste receptors). Future identification of relationship between microcolonies and the healthy host, might permit individualization of therapy, for patients with chronic sinusitis [19]. It will also probably provide more effective treatment or suitable preoperative antibiotic prophylaxis application.

The presence of biofilm could be detected by scanning microscope SEM [20].

In this study, the identification of bacterial film (morphological characteristics of bacteria) was performed and confirmed while using the SEM technique and microbiological identification of the present bacteria. In our study, the presence of plant pollen on the mucosa confirms the aspiration path of bacteria to the sinus lumen (Fig 1C). After the aspiration of microorganisms, the mucous membrane is immediately purified by the ciliary mechanism, which prevents the development of infections [20,21].

The theory on sterility is confirmed, according to some authors, by the identification of bacteria in microbiological testing not exceeding 20% of cases [22–24]. However, not all studies show clear evidence of the microbial sterility of the mucosa. The studies conducted by Brook in 1981 on paranasal sinus mucosa identified both aerobic and anaerobic bacteria [11]. Based on the analysis of swabs taken directly from the sinus or mucous membrane fragments in 1999 Jiang, et al. found that the maxillary sinus mucosa is not devoid of bacteria (46.7% of positive results from swabs, 41.2% of positive results from the sinus mucosa) [25].

Reports in the literature on the state of paranasal sinus mucosa in healthy subjects is limited. The number of procedures during which, there are possibilities non-invasively collecting tissue samples, is very limited. Some environmental studies on the sinuses are based on an analysis of lavage fluid obtained in the puncture of the maxillary sinuses [11,20,22]. Other authors, have studied cultures from swabs or samples taken during endoscopic procedures on the maxillary sinuses, ethmoid [25], sphenoid [26] or frontal sinus [23]. In our case, which was the same as previous studies presented by Cook & Haber and Abou-Hamad, the material was collected from the maxillary sinuses during procedures performed in patients with prognathism [22,24].

In both studies, washes obtained from maxillary sinuses before surgery through an antral puncture were used. They considered that over 80% of specimens from maxillary sinus cavities in asymptomatic adults were sterile [22, 24]. Additionally, Cook & Haber believed that a few bacteria might be present and elicit inflammatory responses [24]. In our studies, we were unable to culture any bacterial strains in only two cases. The results of the microbial culture in comparison with micrographs show differences. It is possible make an attempt to explain this situation by the fact that swabs in previous studies were taken more often outside the mucosa samples than from sinus washes with free planktonic forms. In our studies, swabs were taken directly from sinus mucosa. In total, 41 different microorganisms were isolated. The most frequently found microorganism was *Streptococcus spp*. in over 90% of all samples, while *Propionibacterium acnes* were present in 29.2% of samples, and *Staphylococcus spp*. was present in 17% of the samples. All positive samples had mixed flora.

Local factors that may have an influence on the development of acute or chronic sinusitis include infections with bacteria, viruses, and fungi, defects in mucociliary function, swelling of the sinonasal mucosa, and impairment of normal sinus drainage. Acute sinusitis is often preceded by viral infections [27, 28]. Potentially pathogenic aerobic bacteria are usually responsible for acute sinusitis like *Streptococcus pneumoniae* and *Haemophilus influenza* [29]. In our study among healthy people they were cultured relatively rarely (3% each).

For the development of chronic rhinosinusitis multiple local and general risk factors are responsible, such as allergic rhinitis, defects in immunodeficiency, systemic disorders or anatomic abnormalities causing sinus ostium obstruction. The pathophysiology of chronic sinusitis is uncertain. The theory of chronic infection at this point is controversial, but many studies presented bacterial or fungal biofilm as the main reason for sinus chronic inflammation [30–33].

In patients with impaired immunity, the cause of sinusitis can be opportunistic microorganisms. According to the Centers for Disease Control and Prevention [34] we haven’t isolated any opportunistic bacteria, the only exception was one culture with Candida albicans. In our study group, we didn’t have any patients with a history of systemic disorders or allergic rhinitis. None of the patients have severe anatomical abnormalities in the sinonasal region.

According to *Brook*, the predominant anaerobic bacteria in chronic sinusitis were *Prevotella*, *Porphyromonas*, *Fusobacterium* and *Peptostreptococcus* spp. However, aerobic bacteria like *Staphyloccocus aureus*, *Moraxella catharralis* or *Haemophilus influenzae spp*. may also be responsible for chronic sinusitis [35,36]. Our study shows that the pathogenic bacteria mentioned above are not often present in a healthy person’s culture (*Porphyromonas spp*. *6%*, *Fusobacterium spp*. *3%*, *Peptostreptococcus spp*. *6%*, *Staphylococcus aureus 3%)*.

The host immune system probably is responsible for reducing the risk of infection. The conditions under which bacteria from the biofilm might become pathogenic are unclear and require an explanation.

Mladina, et al. were the first to search for the biofilm on the mucosa of healthy sinuses. The authors [26] took samples from the ethmoid and sphenoid sinuses during neurosurgical procedures of the pituitary gland or orbital decompression in Graves' disease. The authors confirmed the presence of the biofilm in, respectively, up to 94% and 100% of samples from the mucosa of healthy ENT patients. They suggested that the so-called bacterial biofilm is a layer covering the respiratory epithelium, a part of the respiratory mucosal blanket [26]. Containing a large number of pathogenic and inert bacteria, as a product of bacterial flora colonization, it may play a protective role for the sinuses, and its removal can be detrimental. The ability of bacteria strains to form the biofilm structure was studied by Singh, et al. based on culture samples taken from the sinuses of healthy and diseased individuals [37]. The conclusions of their work are confirmed by the presence of the biofilm in the sinus lumen in healthy subjects. They indicate, however, that the biofilm in healthy subjects may be a bacterial colony. Their work was focused mainly to chronic sinusitis and indicate the biofilm as a possible cause of persistent inflammation, antibiotics resistance, and antimicrobial therapy failure [37].

Besides, the swabs were probably taken from planktonic bacteria, which might have affected the results of the subsequent culture. It is also difficult to clearly infer the presence of the biofilm on the basis of a bacteria culture taken from the sinuses, or their characteristics to form the biofilm in vitro.

Contrary to the results of the study conducted by Mladina, et al. [26], we observed that in the maxillary sinus the biofilm is encountered in the form of microcolonies. The bacterial cells show high morphological diversity. Both pathogenic and saprophytic bacteria are involved in the formation of biofilm microcolonies. The biofilm layer does not cover the entire surface of the mucosa. In the maxillary sinuses of healthy subjects, the biofilm is merely an element identified most often on the interface of the layer of the mucosa and cilia.

The presence of bacterial microcolonies in all tested samples, seems to be normal for the mucosal tissue of healthy individuals. In study by Ferrer at the pattern and dynamics of biofilm formation in clinical isolates of *Staphylococcus aureus* and *Staphylococcus epidermidis* in the presence of antibiotics with different action mechanisms were evaluated. Since the biofilm is formed, antibiotic efficacy is significantly reduced, and additionally sub-concentrations of some antibiotics, stimulated biofilm growth [38]. The bacterial microcolonies form may in our opinion explain e.g. limited benefit of antibiotic prophylaxis in uncomplicated implant surgery in healthy patient [39,40].

A strong point of our study was an examination material taken from the inside of sinuses during the surgical procedure in contrast to the studies based on material taken from the middle nasal meatus. The second point was the evaluation of the size and location of the biofilm on the layer of mucous covering healthy mucosa of the sinuses, not reported in the literature yet.

We suspect that the distribution of biofilm, especially development inhibition at the microcolonies form, can be an expression of the host immune response and the mutual interaction between the host and the bacteria.

There is an urgent need to understand special conditions in which biofilm is created *in vivo* in healthy people, which may affect the development of new therapeutic strategies.

While comparing in vitro and in vivo images, it was noted that the in vivo structures of the biofilm are numerous, but have a smaller size (cross-section). No characteristic spatial organization of the biofilm ("mushroom"- like shape) was demonstrated. This may be a response to the host immune response, also resulting from different conditions of the microenvironment and the lack of inhibitory agents in an artificial culture [41]. The structure of the biofilm bound to the surface of the human body is diverse and often difficult to determine [41]. For instance, Aurora Rajeev, et al. observed that the type of microbiome did not differ in healthy subjects and patients with chronic sinusitis (in remission). However, the host immune response was different [42].

The presence of biofilm (regardless of its form) shows that it is not only a momentary accidental contamination of the mucous membranes, but it is the natural form of the bacterial colonization of the mucous membranes of the sinuses. The biofilm is probably in equilibrium under the influence of inhibiting defensive factors of the body, but it can be a source of infection if there are favorable conditions connected with the cellular and humoral immunity, such as general weakening. Unfortunately, not much is yet known about defensive factors which prevent the formation of the biofilm and restrict its development towards chronic infections.

The characteristic for healthy people deployment and small size bacterial microcolonies on the mucous surface require further study. Location bacterial microcolonies on the border of the mucous layer may indicate the permanent process of cleaning the mucosa. This phenomenon could be explained by the theory of specific mucous characteristics in healthy subjects [43].

## Conclusions

In conclusion, the present study increased our knowledge of the presence and size of biofilm on the healthy maxillary sinus mucosa. Our research confirms that the bacteria are dispersed or concentrated in single microcolonies of the biofilm on the border of the mucous covering the ciliary epithelium. There was no uniform layer of the biofilm covering the mucosa of the maxillary sinuses.

Under the conditions of normal homeostasis of the body, the maxillary sinuses present diverse bacterial colonization (a few bacteria, both in the form of the biofilm and planktonic forms). The above-presented results, obtained using SEM, may provide a starting point for the investigation of new approaches in future studies on the mechanism of inhibiting biofilm development in healthy humans and complement research on the microbiota.
